# Are one-stop centres an appropriate model to deliver services to sexually abused children in urban Malawi?

**DOI:** 10.1186/s12887-018-1121-z

**Published:** 2018-04-30

**Authors:** Yabwile Mulambia, Aaron J. Miller, Geraldine MacDonald, Neil Kennedy

**Affiliations:** 1 The College of Medicine, University of Malawi, Chichiri, Blantyre, 3 Malawi; 2 Building Regional Alliances to Nurture Child Health, 2 Gold St, New York, NY 10038 USA; 3 Institute of Child Care Research, Queen’s University, University Rd, Belfast, BT7 1NN UK; 4 Centre for Medical Education, Queens’s University Belfast, University Rd, Belfast, BT7 1NN UK

**Keywords:** Child sexual abuse, Treatment, Program development, Law enforcement, Prosecution

## Abstract

**Background:**

The Republic of Malawi is creating a country-wide system of 28 One-Stop Centres (known as ‘Chikwanekwanes’ - ‘everything under one roof’) to provide medical, legal and psychosocial services for survivors of child maltreatment and adult intimate partner violence. No formal evaluation of the utility of such services has ever been undertaken. This study focused on the experiences of the families served at the country’s first Chikwanekwane in the large, urban city of Blantyre.

**Methods:**

One hundred seven families were surveyed in their home three months after their initial evaluation for sexual abuse at the Blantyre One Stop Centre, and 25 families received a longer interview. The survey was designed to inquire what types of initial evaluation and follow-up services the children received from the medical, legal and social welfare services.

**Results:**

All 107 received an initial medical exam and HIV testing, and 83% received a follow-up HIV test by 3 months; 80.2% were seen by a social welfare worker on the initial visit, and 29% had a home visit by 3 months; 84% were seen by a therapist at the initial visit, and 12% returned for further treatment; 95.3% had an initial police report and 27.1% ended in a criminal conviction for child sexual abuse. Most of the families were satisfied with the service they received, but a quarter of the families were not satisfied with the law enforcement response, and 2% were not happy with the medical assessment. Conclusions: Although a perception of corruption or negligence by police may discourage use of service, we believe that the One-Stop model is an appropriate means to deliver high quality care to survivors of abuse in Malawi.

**Electronic supplementary material:**

The online version of this article (10.1186/s12887-018-1121-z) contains supplementary material, which is available to authorized users.

## Background

With ratification of the new United Nations Sustainable Development Goals, greater attention is being focused on how the medical, legal and social welfare systems should respond in order to ensure the health and safety of these children. Child sexual abuse is increasingly recognized as a public health problem in Sub-Saharan Africa. Of 4412 school children interviewed in a 2005 study in Malawi, 23.8% of children said they had been forced to have sex against their will, 14% reported having been touched on their genitals or breasts against their will, and 3.9% of children over 13 years reported having been forced to engage in some form of oral sex [1]. The Violence Against Children and Young Women Survey in Malawi in 2013 found that 21.8% of girls and 14.8% of boys age 18–24 had experienced sexual abuse prior to age 18; and 37.7% of girls and 9.8% boys age 18–24 said that their first sexual experience before age 18 was unwanted [2]. Services for these children are lacking. Two thirds of sexual assault survivors reported the incident to an authority figure, but only 10% received any professional assistance.

Prior to 2010 in Malawi, the medical, legal and social welfare agencies did not work together to assist survivors of child sexual abuse. Parents would bring their child to police who had not been trained in forensic interviewing or the need for appropriate medical examination. Some of the families were then referred to the hospital for an examination, where only 24% of the doctors could correctly interpret genital findings [3]. In most hospitals they had to wait in separate lines for treatment, HIV testing and emergency contraception. Then they were told to go back to the police with their completed form. The poor linkages between departments led to poor case management and children being lost to follow-up [4–7].

Several countries have developed one-stop centres where medical, legal, social welfare, and counseling services take place under one roof [6, 8]. In the United States, these *child advocacy centers* (CACs) have been shown in prospective, controlled studies to increase police involvement in social welfare cases, increase the number of agencies present during interviews, increase the number of children receiving forensic medical exams, increase the non-offending parent satisfaction with the investigation process, and decrease the percentage of children who are very scared and uncomfortable during the interviewing [6, 7, 9].

In Blantyre, Malawi, the Queen Elizabeth Central Hospital (QECH) is home to the country’s first one-stop centre, referred to as a Chikwanekwane (“everything under one roof”). Building on the work of a multidisciplinary team which has been operating in Blantyre since 2010 [5, 10], the centre was established by funding from DFID with the support of the Ministries of Health, Social Welfare and UNICEF [11]. The team comprises paediatricians, nurses, social workers, police victim support officers and volunteer counselors working together to provide the best care possible for child survivors. Currently, police Victim Support Units (VSU) are the main referral agents, though self-referral is promoted and welcomed. VSU police officers are trained in the provision of counseling and support to victims. At QECH, experienced doctors examine the child and document findings in a medico-legal report. An HIV test and post-exposure prophylaxis is offered, along with follow-up tests at 3 and 6 months after the incident. Sexually transmitted infection management and emergency contraceptives are given when indicated. Social workers and counselors located within QECH offer safety and psychological support respectively. Counselors have longer contact with the victim than any other provider of the service, and arrange to see families weekly as required. Police and social welfare workers then continue their investigation and visit the victim at home to assess the safety of the child.

The anecdotal experience of staff involved in this centre was that it improved client satisfaction and outcomes. As a result, the government of Malawi has rolled out the ‘Chikwanewane’ model to establish 4 other large and 23 smaller centres throughout the country. However, no formal evaluation of the impact of these centres has ever been published from Malawi or, to our knowledge, from Sub-Saharan Africa.

The purpose of this study was two-fold: to explore the experiences of children and families who were provided services at the QECH One-Stop Centre, and to assess the proportion of these families that received medical, legal, and social welfare services according to national guidelines.

## Methods

The participants were families who had received treatment at the Blantyre One-Stop Centre at Queen Elizabeth Central Hospital. QECH is the largest government hospital in Blantyre offering free care to children from the city and Southern Malawi. All children evaluated for sexual abuse during the study period – August 1, 2012 to June 30, 2013 – were eligible. The children’s guardian was the person enrolled in the study and interviewed about their experiences.

The study was introduced to guardians on the day of presentation to the hospital. Guardians were given a brief introductory leaflet explaining the research and contacted 3 months later to determine if they were still willing to be involved in the study. Written consent was obtained. The scope of the questions was designed to assess whether the families had received the medical, legal and social welfare services as required in the Malawi *National Guidelines for Provision of Services for Physical and Sexual Violence* [12]. All interviews took place in the family home 3 months after their evaluation at the One-Stop Centre. All the families received a questionnaire administered in person during this visit (see Additional file 1: Appendix 1). The questionnaire was administered verbally in Chichewa to all the participants; from this cohort, semi-structured interviews were then conducted with 25 guardians who were representative of the study population in terms of age of the victim and gender. With the permission of the respondent, a voice recorder was used to collect the information. The interviews were transcribed. After familiarization of the data in the preliminary analysis, a thematic framework was developed and themes coded. The researchers were blinded to the child protection services outcomes at the time of the visit. The child’s guardian was interviewed in order to prevent secondary victimization of the survivor.

Inclusion criteria: Participation in this study was offered to the guardians of all children age 0–14 years who, in the opinion of the medical staff, had been sexually abused and who gave consent to be in the study. From this cohort of participants, a representative subset of 25 guardians were chosen to also participate in the semi-structured interview. Exclusion criteria: cases in which the guardian did not give consent, or in which the final diagnosis was not sexual abuse.

## Results

**Fig. 1:**
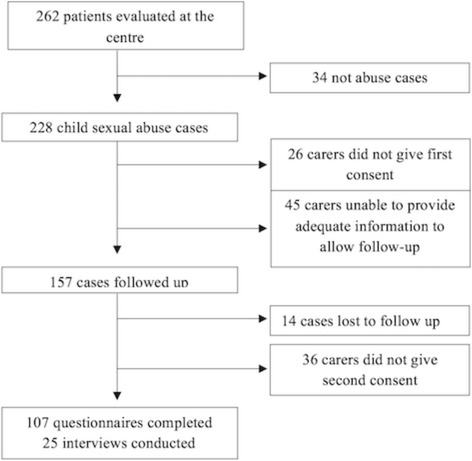
Consort diagram of Study Participants

In total, 262 patients were evaluated at the Queen Elizabeth Central Hospital One-Stop Centre between August 1, 2012 and June 30, 2013 (see Fig. 1). Ten cases of physical assault were removed from analysis. An additional 24 cases were removed as the allegation of abuse was not substantiated, and the presentation was unrelated to child sexual abuse. Of the remaining 228 child abuse survivors and their guardians, 59 were lost to follow up and 62 did not give consent, leaving 107 participants who completed the questionnaire. Semi- structured interviews (SSI) were held with 25 guardians in their homes and with 10 service providers representing all the agencies involved in the child protection service.

Three of the questionnaires were conducted either at the centre or another site other than home because the perpetrator or relatives of the perpetrator where at home and guardians felt the environment was unsafe. Ninety-nine per cent of the children were girls, average age 9 years (see Table 1).

**Table 1:** Participant Demographic Data, *n* = 107

Children Characteristics	Frequency
Female	99.1%
Mean Age (SD)	9.4(3.9)
Guardian Characteristics	Frequency
Female	75.7%
Mean Age (SD)	36.1(9.8)
Average Household size	5.8
Level of Education	
No Education	8.4%
Primary School	43.9%
Secondary School	37.4%
Tertiary	10.3%

### How was the abuse discovered, and why did people come?

Most of the children in this sample (57.9%) disclosed the sexual abuse on their own (“spontaneously”) without being prompted or having the abuse witnessed by a concerned 3rd party (see Table 2).

**Table 2:** Pattern of Disclosure

Pattern of disclosure	Frequency	Percentage
Spontaneous	62	57.9
Witnessed assault	25	23.4
Prompted	12	11.2
Informed by witness	8	7.5

Most of the semi-structured interview participants (19 of 25) sought care for their children due to the fear of HIV infection (see Table 3). Other reasons included the desire to “verify” if the child was raped (5 of 25), the desire for justice (3 of 25), for counseling (2 of 25), pregnancy (1 of 25), and just because they were referred by police (1 of 25). Most participants expected an HIV test, physical examination, HIV Post-Exposure Prophylaxis (PEP) and justice. Very few mentioned counseling services and none mentioned social services as a reason for seeking help.

**Table 3:** Sample quotes from semi-structured interview participants on why they sought services at the one-stop centre

Risk of HIV infection: “What we really wanted to find out was the HIV status of the child since there is HIV/AIDS because this is what we fear the child has contracted.”	
Justice: “Having justice done would have been a lesson to other people.”	
Psychological support: “This was affecting her, she was depressed and not eating. After school she went straight to her bedroom and wanted to be alone… I found it abnormal.”	
Fear of pregnancy: “First of all, I feared she could be pregnant; she could fall unexpectedly. Secondly, there are sexually transmitted diseases...”	

### Performance according to the Malawi National Guidelines for provision of services to survivors of violence

**Fig. 2:**
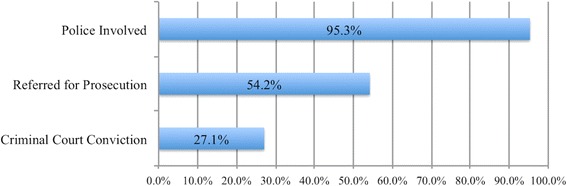
Law Enforcement Outcomes, *n* = 107

Seventy-two of 107 survivors (67.3%) arrived within 72 h in time to start HIV Post-Exposure Prophylaxis (PEP). All received appropriate medical services. 80% had a follow up visit for a second HIV test at 3 months (see Table 4). The requirement for Social Welfare is for an initial assessment at the Chikwanekwane, followed by a home visit within 3 months. The initial assessment by Social Welfare was performed in 80% of the cases during the initial visit at the One-Stop Centre, but only 29% of the children had a home visit due to a lack of transport. Eighty-four percent of children received psychological support during the initial visit, In 95% of cases there was some form of police investigation with 27.1% of the cases resulting in criminal conviction (Fig. 2).

**Table 4:** Performance by Each Agency, *n* = 107

Adequately handled cases (Indicator)	At Presentation %	By 3 months (%)
Health Examination, HIV 0-3 months)	100	82
Social (Initial assessment, home visit by 3 months	80.2	29
Psychological (Support offered to all clients)	84	12 more than one visit
Justice (Investigation,referral to court)	95.3	53.8

Overall, 18% of the child abuse cases received all the services required according to the national guidelines.

Of the 107 families, 79 (73.8%) were satisfied with the services provided, and 28 (26.2%) were not satisfied (see Table 5 and Table 6). The commonest reason was dissatisfaction with police with concerns regarding perceived corruption and negligence.

**Table 5:** Evaluation of Service Satisfaction

26.2% (28/107) were not satisfied with the service	Total
Police	
Perceived corruption (9)	21
Perceived Negligence (3)	
Court: Prison sentence too short	4
Medical Services	
HIV post-exposure prophylaxis not provided (1)	2
Medical exam took too long (1)	
Counseling: Wanted more sessions	1

**Table 6:** Sample quotes from semi-structured interview participants on their dissatisfaction with one-stop centre services

Perceived inaction of police: “Then they (the police) said if you can manage to catch the perpetrator, bring him here but if you don’t have a means of doing that we will see what we can do.”	
Lack of professionalism and capacity: “The first hearing in court, the prosecutor did not come. Other police officers said he was on leave then. We were told to come on another date. The next scheduled court hearing, he didn’t show up again. They said he was sorting out his family issues.”	
HIV post-exposure prophylaxis not given: “Nothing! We were not given anything…I really hope she continues to remain HIV negative”	
Family member perpetrator: The reason the case was not taken to court was that, I am the last born in our family and my elder sister’s son is the one who did this...There is no peace in our family now.	

No statistically significant associations were found between client satisfaction levels, and receipt of services (health, social welfare, police or counseling) or outcomes of the case (case referred to court or conviction of the perpetrator) (Table 7).

**Table 7:** Caregiver satisfaction according to receipt of services provided (*n* = 107)

		Expressed satisfaction with the overall service	Expressed dissatisfaction with the overall service	Fishers exact test
Agency involvement at presentation	Health – got HIV test and PEP at presentation	52	17	0.81
	Health – did not get HIV test *OR* got HIV test but not PEP at presentation	25	9	
	Justice-got service	30	8	0.64
	Justice-did not get service	49	18	
	Social –got service	64	20	0.77
	Social –did not get service	15	6	
	Psychological –got service	66	22	1.00
	Psychological-did not get service	13	4	
Case outcomes	Perpetrator convicted	25	4	0.13
	Perpetrator not convicted	54	22	
	Case referred to court	43	14	1.00
	Case not referred to court	36	12	

#### Impact of child sexual abuse on the child and family

The psychological impact of child sexual abuse was evident to guardians with older children. The semi-structured interview participants expressed feelings of frustration, fear and disbelief (never imagined it would happen to their child). Furthermore, the problem of stigma came up with participants concerned about community perspectives of child sexual abuse, which was often misinterpreted as consensual sex especially if the child involved is older (see Table 8). The child is perceived as immoral and discriminated in the community. Disruption of family relationships was reported when the perpetrator was a family member with some reporting mothers neglecting their child’s welfare to save their marriage when the perpetrator was the breadwinner.

**Table 8:** Sample quotes from semi-structured interview participants on the effects of child sexual abuse on the family

Emotional and psychological impact	
“After the incident, she now wets and soils the bed…where she sleeps is a sorry sight.” Guardian (*mum shedding tears*)	
Stigma	
“The challenges that she is mainly facing is that she is being discriminated. For example, she will go to her friend’s place and her friend’s mother says to her you should not be chatting with my child because you have immoral behaviour” Guardian	
Effects on family relationships	
“She was being abused by her step father…she told her mum first but she did nothing. She accused her of trying to break her marriage…. She (victim) last saw her mother in court. Until today, her mother has no interest in the welfare of the child.” Guardian	

## Discussion

This is the first study from within the region or a low-income setting to evaluate and report the services provided by a one-stop centre for survivors of child sexual abuse. Building the one-stop centre at Queen Elizabeth Central Hospital in 2012 was associated with nearly tripling the number of children receiving services of sexual abuse each year: from 121 to 168 per year (2006–2011) to 356 in 2014. The majority of clients received the services they needed at presentation, but follow-up by social welfare was hindered by lack of funding. Overall, we found very high levels of client satisfaction, particularly with medical and social welfare services. Despite over half of cases resulting in a court case, and 27% in a successful prosecution, the commonest reason for reporting dissatisfaction was concerns regarding the performance of police. Satisfaction levels were not significantly associated with receipt of services or the outcome of the case. This may be a reflection of the sample size, or because receipt of one service compensated for the lack of receipt of another.

We do not know who referred each child to the one-stop centre, but the fact that 67% of the children arrived in time for HIV post-exposure prophylaxis is encouraging and worth further study. Fear of HIV was the strongest motivator for seeking services (76%), and the hospital had the highest follow-up rate (82%) of all the agencies. In a country with a high HIV prevalence of 10.0% [13], the one-stop centre system could be an effective point for HIV prevention, which not only saves the survivor from a life of uncertainty and stigma, but also saves the healthcare system the future costs of HIV care.

A high proportion of our clients in this sample were female, reflecting the profile of service users at the time of the study. In a country where homosexuality remains illegal, male survivors of sexual violence face significant stigma that limits disclosure.

We found a relatively high prosecution rate (27.1%). Past research in industrialized countries has also demonstrated increased prosecution rates and quicker case resolution in districts where medical, legal and social welfare are co-located together [14, 15]. Our study was not designed to assess how the 58 cases referred for prosecution were different than the 49 cases which were not referred for prosecution; nor did we attempt to assess what case characteristics were associated with conviction versus acquittal. The 3-month time frame was deemed adequate to assess whether criminal court cases would reach conclusion based on discussions with local magistrates, prosecutors and police who stated that most cases are resolved – either dropped or move forward - within a few weeks. Very few respondents have attorneys to represent them in court, and almost all cases are closed in less than 3 months.

We acknowledge several limitations to our study: of the 228 children diagnosed with sexual abuse, 60 of the families (26.3%) did not agree to be part of the study, which may represent dissatisfaction with services; we did not interview the child (which may have yielded different answers); and self-report surveys, in general, provide only the participants’ perceptions – it is unknown if there were 9 actual cases of police corruption or whether there was a valid reason the case did not move forward which was not communicated to the family. Furthermore, our study was limited to an urban area and the findings can not necessarily be generalized to rural settings.

Our study also had several strengths. It is a relatively large series of client responses from this type of service and provides valuable insight into the experience of families involved. In addition, to a large extent the results obtained from both the qualitative and quantitative components concurred with each other, suggesting that they were reliable.

With our new understanding of the numerous deleterious short and long-term effects of abuse, and how it leads to a high disease burden on society [16], it was essential that 6 of the new subgoals in the UN Sustainable Development Goals address violence against children and intimate partner violence. Bringing together medical, legal and social welfare agencies - and continuing to develop their capacity - will be key components to ensuring that we interrupt the cycle of violence as early as possible and that we work toward effective solutions for primary and secondary prevention of violence so that every child can grow up with safe, stable, nurturing relationships. We believe that the one-stop centre approach offers a useful model for providing such services in a low-income urban setting.

## Conclusion

One-stop centres where health practitioners, police and social workers coordinate closely, was an effective strategy to improve the health, safety and well-being of survivors of sexual and physical violence in an urban setting in Malawi. Longer term evaluation studies which listen to the voice of survivors in this and other urban African settings are needed. Effective service models for rural survivors of violence are required.

## Additional file

Additional file 1: Appendix. Contains the questionnaire and interview guide used to collect data. (PDF 75 kb)

## Abbreviations

HIV: Human Immunodeficiency Virus
OSC: One Stop Centre
PEP: Post-Exposure Prophylaxis
QECH: Queen Elizabeth Central Hospital
SD: Standard Deviation
SSI: Semi-structured Interview
UN: United Nations
UNICEF: United Nations Children’s Emergency Fund
VSU: Victim Support Unit

## References

[1] Burton P (2005). Suffering at school: the results of the Malawi gender-based violence in schools survey.

[2] Violence against children and young women in Malawi (2014). Findings from a national survey. Ministry of Gender.

[3] Miller A, Toombs K (2009). Educating physicians internationally in the diagnosis of child sexual abuse: Evaluation of a brief educational intervention in Malawi. J Child Sex Abus: Research, Treatment, & Program Innovations for Victims, Survivors, & Offenders.

[4] Richter L, Dawes A, Higson-smith C. Sexual abuse of young children in Southern Africa. Cape Town: HRSC press; 2005.

[5] Chesshyre ELD, Molyneux EM (2009). Presentation of child sexual abuse cases to queen Elizabeth central hospital following the establishment of an HIV post-exposure prophylaxis program. Malawi Med J.

[6] Jones LM, Cross TP, Walsh WA, Simone M (2007). Do Children’s advocacy centers improve families’ experiences of child sexual abuse investigations?. Child Abuse Negl.

[7] Walsh WA, Cross TP, Jones LM, Simone M, Kolko DJ (2007). Which sexual abuse victims receive a forensic medical examination? The impact of Children’s Advocacy Centers. Child Abuse & Neglect.

[8] Allnock D (2009). Sexual abuse and therapeutic services for children and young people: the gap between provision and need.

[9] Cross TP, Jones LM, Walsh WA, Simone M, Kolko D (2007). Child forensic interviewing in Children’s advocacy centers: empirical data on a practice model. Child Abuse Negl.

[10] Mtibo C, Kennedy N, Umar E (2011). Explanations for child sexual abuse given by convicted offenders in Malawi: no evidence for “HIV cleansing”. Child Abuse Negl.

[11] Molyneux EM, Kennedy N, Dano A, Mulambia Y (2013). Sexual abuse of children in low-income settings: time for action. Paediatrics and International Child Health.

[12] National Guidelines for Provision of Services for Physical and Sexual Violence (2015). Malawi ministries of health, Gender.

[13] UNAIDS. http://www.unaids.org/en/regionscountries/countries/malawi. Accessed on 25 Nov 2015.

[14] Miller A, Rubin D (2009). The contribution of children’s advocacy centers to criminal prosecutions of child sexual abuse. Child Abuse & Neglect: The International Journal.

[15] Walsh WA, Lippert T, Cross TP, Maurice DM, Davison KS (2008). How long to prosecute child sexual abuse for a community using a children’s advocacy center and two comparison communities?. Child Maltreatment.

[16] Reza A (2009). Sexual violence and its health consequences for female children in Swaziland: a cluster survey study. Lancet.

