# Supplementary material for: Are one-stop centres an appropriate model to deliver services to sexually abused children in urban Malawi?
Source: BMC Pediatr. 2018 Apr 30;18:145. doi: 10.1186/s12887-018-1121-z (PMC5925825; doi:10.1186/s12887-018-1121-z)
Supplement: Supplementary file 1 — Appendix. Contains the questionnaire and interview guide used to collect data. (PDF 75 kb) [file 12887_2018_1121_MOESM1_ESM.pdf]

APPENDIX

Appendix 1: QUESTIONNAIRE FOR GUARDIANS

Date of interview________________ _______ ID number__________

1. Age of child in years……………………………………………………………..
2. Sex of child a)male □ b)female □
3. Age of guardian in years…………………………………………………………..
4. Sex of guardian a)male □ b)female □
5. Level of education attained by guardian
   a. Primary □
   b. Secondary□
   c. Tertiary □
6. Relationship with the victim (eg Mother, aunt etc)

   ………………………………………………………………………………..

7. How many people live in this household? Number □
8. How did you find out about the incident? (Who disclosed this to you?)
9. Who made the decision that the child should be taken for care after the incident?
   a. Mother□
   b. Father□

c. Other relative□

d. Friend □

e. Other – specify□ ……………………………………

10. When did you come to hospital after the incident ?in days

		a)Yes	b)No
	Did you go to the police after the incident?		
	Did you come to the hospital for a follow up visit at 3 months?		
	Did you meet social worker?		
	Did you have follow up home visits by CCPW or social workers?		
	Did you meet the counselors?		
	How many times did you meet the counselors?		
	Were you referred to court?		
	Are the police investigating the case?		
	Was the perpetrator prosecuted		

20. Did the hospital do an HIV test on the first day of presentation, and at 3 months?

21. Did the hospital provide PEP? If not, do you think they should have?

22. How did you get to the hospital? How much did it cost? Could you afford it?

23. Who was the abuser? (if known – move on to q24)

24. Did the identity of the abuser make it easier or harder for you to seek help for your child?

25. Anything else you'd like to say about the service you received for your child? Free text

Appendix 1a: MAFUNSO PA MAGANIZO A WOYANG'ANIRA

Tsiku ___________________ Nambala _________

1. Zaka za Mwana ……………………………..

2. Mwana wanu ndi a)Wamwamuna □ b)Wamkazi □

3. Zaka za kholo/womung'anira …………………………………

4. Woyang'anira ndi a)Wamwamuna □ b)Wamkazi □

5. Mulingo wamaphunziro umene muli nawo

   a. Pulayimale □

   b. Sekondale□

   c. Kupitirira sekondale □

6. Ubale ndi mwana wokhuzidwayo……………………

(chitsanzo; Azakhali mayi, bambo)

7. Ndi anthu angati amene amakhala nyumbamu? Wokwana □

8. Munadziwa bwanji za zimene zinachitikazo? Amene anakuwuzani ndi ndani?

   a. Mwanayo-mwini wake

   b. Ndinachita kumufunsa

c. Munthu wina wapadera amene anawona

d. Munthu wina wapadera sanawone koma anachita kuwuzidwa

9. Ndi ndani amene anapanga chiganizo kuti mwanayu ayenera kupita kukalandira chisamaliro izi zitachitika?

a. Amayi ake

b. Abambo ake

c. Abale ena

d. Mzanga

10. Kodi munapita kuchipatala patatha masiku angati zitachitika izi ?

		a)Eya	b)Ayi
	Kodi munapita ku polisi zitachitika izi?		
	Kodi munabwera ku chipatala patapita miyezi itatu kuti adzakuwoneni?		
	Kodi munakumana ndi a social worker?		
	Kodi a social worker anakuyendelani ku nyumba mutachoka kuchipatala.		
	Kodi munakumana ndia khansala ku chipatala?		
	Kodi a khansala munakumana nawo kangati ?		
	Nanga anakuwuzani kuti mupite ku bwalo la milandu?		
	Kodi apolisi akufufuzabe za nkhaniyi?		
	Kodi Munthu anachita izi analandira chilango?		

20. kodi anamuyeza magazi kuchipatala kuti adziwe ngati ali ndi kachilombo koyambitsa matenda a EDZI mutaonedwa koyamba?.........................

Nanga patatha miyezi itatu………………………

21 Nanga akuchipatala anapeleka mankhwala omuteteza mwana kuti asatenge kachilombo koyambitsa matenda a Edzi zitachitika izi?

Ngati sanapatsidwe mankhwala inu mukuona ngati anayenela kutero?

22. Kodi Munayenda bwanji kupita kuchipatala? Munalipila ndalama zochuluka bwanji? Nanga inu munakwanitsa kulipila popanda vuto lililonse?

23. Kodi anachita izi mukumudziwa?(ngati akuziwika, funsani 24)

24. Ngati mukumudziwa, kodi izi zidapangitsa kuti chikhale chovuta kapena chosavuta kupanga ganizo lopeza chithandizo cha mwana wanu?

25. Muli ndi mau onjezela okhudza chithandizo chomwe mwana wanu walandila ?

Appendix 2: **<u>SEMI-STRUCTURED INTERVIEW GUIDE - GUARDIAN</u>**

1. Why did you seek care when you found out your child has been sexually abused?
    a. What prompted you to come?
    b. Who made the decision that the child should be brought to the hospital?
    c. When after the incident did you bring your child for help? in days
2. Were all family members in support of child been brought to the hospital? If not who and why?
3. What sort of help where you expecting or looking for?
4. Do you think the current services helped you? (go through each service in turn – medical, social welfare, police and prosecution, counselors)

a. What is their strength?

b. What is their weakness?

c. Are they harmful, if so how?

5. How could the service you experienced be improved?

6. Anything else you'd like to say about the service you received for your child?
